# Cross-sectional prevalence survey of intimate partner violence perpetration and victimization in Canadian military personnel

**DOI:** 10.1186/1471-2458-13-1019

**Published:** 2013-10-28

**Authors:** Mark A Zamorski, Miriam E Wiens-Kinkaid

**Affiliations:** 1 Department of Family Medicine, Faculty of Medicine, University of Ottawa, Ottawa, Canada; 2 Directorate of Mental Health, Canadian Forces Health Services Group Headquarters, 1745 Alta Vista Dr, Ottawa, ON, Canada; 3 Directorate of Force Health Protection, Canadian Forces Health Services Group Headquarters, 1745 Alta Vista Dr, Ottawa, ON, Canada

**Keywords:** Spouse abuse/statistics and numerical data, Military personnel, Marital relationship, Marital conflict, Mental disorders, Stress disorder, Post-traumatic, Depression, Alcohol consumption, Cross-sectional study, Occupational health

## Abstract

**Background:**

Intimate partner violence (IPV) is prevalent and is associated with a broad range of adverse consequences. In military organizations, IPV may have special implications, such as the potential of service-related mental disorders to trigger IPV. However, the Canadian Armed Forces (CAF) have limited data to guide their prevention and control efforts.

**Methods:**

Self-reported IPV perpetration, victimization, and their correlates were assessed on a cross-sectional survey of a stratified random sample of currently-serving Canadian Regular Forces personnel (N = 2157). The four primary outcomes were perpetration or victimization of any physical and/or sexual or emotional and/or financial IPV over the lifespan of the current relationship.

**Results:**

Among the 81% of the population in a current relationship, perpetration of any physical and/or sexual IPV was reported in 9%; victimization was reported in 15%. Any emotional and/or financial abuse was reported by 19% (perpetration) and 22% (victimization). Less physically injurious forms of abuse predominated. Logistic regression modelling showed that relationship dissatisfaction was independently associated with all four outcomes (OR range = 2.3 to 3.7). Probable depression was associated with all outcomes except physical and/or sexual IPV victimization (OR range = 2.5 – 2.7). PTSD symptoms were only associated with physical and/or sexual IPV perpetration (OR = 3.2, CI = 1.4 to 7.9). High-risk drinking was associated with emotional and/or financial abuse. Risk of IPV was lowest in those who had recent deployment experience; remote deployment experience (vs. never having deployed) was an independent risk factor for all IPV outcomes (OR range = 2.0 – 3.4).

**Conclusions:**

IPV affects an important minority of military families; less severe cases predominate. Mental disorders, high-risk drinking, relationship dissatisfaction, and remote deployment were independently associated with abuse outcomes. The primary limitations of this analysis are its use of self-report data from military personnel (not their intimate partners) and the cross-sectional nature of the survey. Prevention efforts in the CAF need to target the full spectrum of IPV. Mental disorders, high-risk drinking, and relationship dissatisfaction are potential targets for risk reduction. Additional research is needed to understand the association of remote deployment with IPV.

## Background

Intimate partner violence (IPV) is an important global public health problem [1]. In 2009, 6% of Canadians reported having been physically or sexually victimized by a current or past spouse over the previous five years [2]. Considered to be another form of IPV, emotional and/or financial abuse (e.g., name calling, preventing access to family income [1]), is far more prevalent, affecting 17% of Canadians over the previous five years [2].

IPV is associated with a host of negative effects, including serious physical injuries, mental disorders, and drug and alcohol abuse [1]. Annual direct and indirect costs of IPV in Canada amount to billions of dollars [3]. Exposure to IPV as a child is associated with a broad range of negative physical and mental health outcomes over the entire life course [4]; those so exposed are also significantly more likely to experience or perpetrate IPV in adulthood [4]. A broad range of risk factors for perpetration of IPV have been reported [1,5], including male sex (for sexual and more severe forms of physical IPV, at least), youth, unemployment, low income, heavy alcohol consumption, certain mental disorders (including post-traumatic stress disorder (PTSD) [6]), family conflict, and low social support.

IPV certainly occurs in military families [7,8], where it potentially has special significance [9]. For example, the prevalence of IPV could be different from that of the general population [8]. Factors favoring perpetration of IPV by military personnel could include their male predominance, their relative youth, and higher risk of heavy alcohol consumption (with the latter being seen in some [10,11] but not all [12] military organizations. Protective factors in military personnel could include employment, higher family income, and lower rates of hard drug use [8]. As well, aspects of military life, such as frequent relocation and separation required by military duties, can add to family stress and strain, erode social support, and constrain employment prospects for intimate partners [13]; these could increase the likelihood of IPV, intensify its negative effects, or make it more difficult for intimate partners to leave an abusive relationship. Military culture, notably its male dominance and its use of violence as a legitimate tool to settle conflicts, might also play a role [14,15].

In addition to differences in prevalence in IPV relative to the general population, there may be differences with respect to its phenomenology (e.g., severity [16], frequency, duration, barriers to help-seeking). As an employer, military organizations are concerned with occupational impacts of IPV [17-19], e.g. absenteeism, productivity losses, and increased health care expenditures for employees and dependents [20]. Finally, military organizations may also have special opportunities and obstacles when it comes to prevention of IPV [9].

Canada has deployed more than 40,000 personnel in support of its combat and peace support mission in Afghanistan, and particular attention has been focused of late on the potential association of combat deployments with perpetration of spousal violence [6]. The link of combat deployments with mental disorders [21] and the latter’s association with perpetration of IPV [6] presents a plausible mechanism for this association. In addition, failure to change behaviors that are adaptive in a combat zone but destructive at home (e.g., targeted use of aggression [22]) could also contribute. If so, a higher risk in the immediate post-deployment period would be expected. Data on the linkage between deployment and IPV are limited, with some [23,24] but not all [25] studies showing a small increased risk in perpetration of more severe physical IPV after deployment.

The Canadian Armed Forces have a comprehensive prevention program for IPV and other forms of family violence [9]. Its family violence policy requires yearly training on family violence for all commanding officers. Since 2007, there has been a CAF-wide Family Violence Awareness Campaign, which sensitizes CAF families to family violence as a problem and aims to overcome barriers to care. Intervention for family violence cases are coordinated by multi-disciplinary Family Crisis Teams at each base. The CAF have a broad range of risk factor reduction programs for drivers of family violence such as mental disorders, high-risk drinking, family conflict, and social isolation. Prevention efforts are guided and coordinated by their Family Violence Advisory Committee. In response to public concerns over the CAF’s response to family violence, the Surgeon General empanelled the Canadian Armed Forces Expert Panel on the Prevention of Family Violence in January 2012. While the Panel found the CAF’s prevention approach to be consistent with best practices, room for improvement in certain areas was identified (notably, in the areas of governance, policy, and accountability surrounding family violence [9]).

Notwithstanding these efforts and other factors that might change the prevalence or other aspects of IPV relative to civilians, very little has been known about the prevalence, severity, impacts, and correlates of IPV in the Canadian Armed Forces [14]. Data from other military organizations may not apply, due to differences in the military population, the particular stresses and strains faced by military families, the supports and services available, and the overall preventive strategy used by each military organization. This knowledge is essential in order to develop and evaluate prevention efforts. Accordingly, the objectives of this study are as follows:

•Describe the prevalence and severity of the full range of IPV perpetration and victimization in Canadian military personnel; and

•Explore the correlates of IPV, including sex, age, rank, deployment history, mental disorders, heavy alcohol consumption, and relationship satisfaction, with the goal of identifying risk groups and potential contributing factors.

## Methods

### Study design

The study was a cross-sectional, population-based survey of currently-serving Canadian Regular Forces personnel.

### Data source

Data from the CAF’s 2008/2009 Health and Lifestyle Information Survey (HLIS) [12] was used for this study. HLIS is the CAF’s periodic health surveillance survey; its sample consisted of a random sample of Canadian Armed Forces personnel in the Regular Forces component on 28 July 2008, stratified by gender, rank, and recent deployment history. The survey was accurately framed as a general health surveillance survey, not a survey specifically on IPV; no compensation was offered for completion of the survey. Reservists and personnel who were not considered part of Total Effective Strength (e.g., those on long-term sick leave, those in basic training) were excluded. Potential participants were identified using the Canadian Armed Forces’ Human Resources Management System. The target sample size was 1922 respondents, which was determined using the exact method in order to yield error estimates of +/− 3%. The anonymous paper survey was mailed to 4744 potential respondents over a one-year period starting in November 2008; two reminders were used for non-respondents. After adjustment for valid reasons for non-response (e.g., the respondent had released from the Regular Force), 2315 out of 4385 potential respondents returned surveys, representing a 49% gross response rate and a 53% cooperation rate. There were at most small degrees of under- and over-representation of sociodemographic groups in the sample, and this was accounted for in the sample weights. After exclusion of 158 unusable surveys (almost entirely due to incomplete sociodemographic data for calculating sample weights), 2157 surveys were available for analysis. This analysis of IPV was limited to the 1745 respondents (81% of target population) who responded affirmatively to the question “Are you currently in a relationship?”

### Survey instrument

#### Intimate partner violence

For the purpose of this paper, intimate partner violence was considered to include intentional physical, sexual, or psychological harm (to include financial abuse, such as limiting access to family income) by a current spouse or intimate partner. IPV was assessed in those currently in a relationship using items based on those used in the Canadian General Social Survey (GSS) on Victimization [2]. The GSS IPV items have been used in national Canadian surveys since the 1993 Violence Against Women Survey; the specific violent acts included were intended to cover the full spectrum of criminal violence under Canada’s *Criminal Code*[26]. The items were selected and adapted [27] from the most widely used and best validated survey instrument for measuring IPV, the Conflict Tactics Scale [28]. The questions differed from those in the GSS in that they also examined perpetration of IPV; they also used a different recall period for abuse occurrence (over the life of the current relationship as opposed to the previous five years used in the GSS). Another difference is that the present survey assessed IPV only with their current intimate partner as opposed to a current *or* past partner as was done in the GSS.

For physical and sexual IPV, respondents indicated whether 10 acts of abuse occurred between them and their current spouse/partner over the entire time of that relationship. For each abuse act listed, respondents indicated whether they were a victim and/or a perpetrator of the abuse. The acts of abuse were the same as in the GSS and ranged in severity from threats of being hit to forced sexual activity. Similarly, emotional and financial IPV was measured over the entire course of the current relationship for seven abuse acts. Examples of emotional and/or financial acts included limiting contact with family/friends and preventing access to family income.

The current analysis used four aggregated IPV variables as primary outcomes: any physical and/or sexual IPV perpetration, any physical and/or sexual IPV victimization, any emotional and/or financial IPV perpetration, and any emotional and/or financial IPV victimization.

#### Socio-demographic and military characteristics

Participants responded to questions on sex, age category, military rank category, and overseas deployment history. For this analysis, respondents were categorized by deployment history into three groups based on the recency of an operational deployment (never deployed, recent deployment (within the previous two years), and remote deployment (more than two years previously)).

#### Mental disorders, high-risk drinking, and relationship satisfaction

Probable depression was assessed using the depression component of the World Health Organization’s Composite International Diagnostic Interview Short-form (CIDI-SF), using a cut-off of five or more symptoms of depression, which indicates a 90% probability of an episode of major depression in the previous 12 months [29]. Current symptoms of PTSD were assessed using the four-item Primary Care PTSD Screen, using a cut-off of three or more positive responses to the four yes-no items in the scale; this cut-off is 78% sensitive and 87% specific for PTSD in primary care populations [30]. Hazardous and harmful drinking (hereafter labelled “high-risk drinking”) was assessed using the Alcohol Use Disorders Identification Test [31], using a cut-off of eight or above for men and seven and above for women (possible range = 0 to 40) [32]. Relationship satisfaction was determined using a single item taken from the evaluation of a military marital education program [33]: “Please use this rating scale to indicate how satisfied you are in your relationship…” Response categories were: “extremely satisfied,” “very satisfied,” “somewhat satisfied,” “mixed or unsure,” “somewhat unsatisfied,” “very dissatisfied,” and “extremely dissatisfied.” For this analysis, those with scores more favourable than “somewhat satisfied” were considered “well satisfied”; others were considered “not well satisfied.”

### Analysis

Stata version 12.1 for Windows was used for analyses. To account for the survey’s complex sampling strategy, all weighed estimates were produced using Stata’s survey data analysis functions. Prevalence estimates are reported with their 95% confidence intervals (CI); standard errors were calculated using a linearized variance estimator based on a first-order Taylor series linear approximation. Cells with fewer than 20 responses were suppressed. Bivariate (unadjusted) analyses examined the associations between the four IPV outcomes and other variables of interest using logistic regression. Logistic regression was also used to explore the independent association of different forms of IPV perpetration and victimization with potential predictors; these are expressed as adjusted odds ratios (OR) and their associated 95% CI.

The amount of missing data for each variable ranged from 0 to 12%. The greatest degree of missingness was seen for the hazardous and harmful drinking (12%), relationship satisfaction (6%) and probable depression (3%) variables; all other variables including the four summary abuse variables, had fewer than 3% missing observations. Multiple imputation using chained equations was used in the final multivariate models. The imputation model included all the variables in the final model as well as the stratum and weight variables to account for the complex survey design; 150 imputations were completed. Due to a small amount of missing data in predictors of the imputation model (between 3 and 9 observations per variable) a total of 1729 unweighted observations out of a possible 1745 were used in the models. Prior to imputation, goodness of model fit was assessed using the method described by Archer & Lemeshow which allows examination of model fit while using survey data with complex sampling [34], and multicollinearity was examined, taking into account the survey design, with a tolerance and variance inflation factor (VIF) using a cut-off of VIF > 10 to indicate multicollinearity [35].

### Ethical aspects

Completion of the survey was voluntary and anonymous. The HLIS was approved by an independent research ethics board (Ethica Clinical Research, Montreal, Quebec).

## Results

### Description of respondents

The socio-demographic and military characteristics of the population of CAF Regular Force personnel who reported currently being in an intimate relationship are shown in Table 1. The population consisted largely of young and middle-aged men with substantial military experience. 67% had had a previous overseas deployment during their career, with 43% having deployed only more than two years previously and 24% having deployed in the previous two years. Relationship satisfaction (Table 2) was high: 75% were “very” or “extremely” satisfied with their current relationship; only 4% were overtly dissatisfied. Probable depression, PTSD symptoms, and high-risk drinking were seen in 7.1%, 7.3%, and 18.0% of the population, respectively (Table 2); 27.3% of the population had one or more of these problems. Co-morbidity of probable depression and PTSD symptoms was common, with 4% of the population having both conditions, 5% having only PTSD symptoms, and 6% having only probable depression.

**Table 1:** Socio-demographic and military characteristics of the population currently in an intimate relationship (N = 1745)*

**Variable**	**Value**	**N (unweighted)**	**% (weighted)**
Sex	Male	1017	87.81
	Female	728	12.19
Age category	18-29	313	23.69
	30-39	583	29.77
	40-60	849	46.54
First official language	English	1167	71.44
	French	570	28.56
Marital status	Married	1137	66.31
	Not married	600	33.69
Years of service	<5	146	11.21
	5-14	621	33.70
	≥ 15	978	55.09
Element	Land	824	44.79
	Sea	272	17.44
	Air	645	37.76
Rank (at time of survey)	Junior NCM^†^	488	47.42
	Senior NCM^†^	302	29.50
	Officer	955	23.08
Deployment	Never	452	33.09
	Remote	468	42.68
	Recent	824	24.23

*Counts and percentages presented reflect values prior to imputation.

†Junior NCM = non-commissioned member at rank below Sergeant or the equivalent; Senior NCM = non-commissioned member at rank of Sergeant (or the equivalent) or above.

**Table 2:** Mental health problems, high-risk drinking, and relationship satisfaction (N = 1745)*

**Characteristic**		**n/N (unweighted)**	**n/N (weighted)**	**% (95% CI)**
Mental health	Probable depression (CIDI-SF)	123/1688	3267/46232	7.1 (5.0–9.1)
	PTSD symptoms (PC-PTSD)	113/1741	3478/47815	7.3 (5.0–9.5)
Substance use	High-risk drinking (AUDIT)	248/1551	7548/42025	18.0 (14.7–21.2)
Relationship satisfaction	Well satisfied^†^	1252/1650	33179/44281	74.9 (71.4–78.5)
	Extremely satisfied	593/1650	15623/44281	35.3 (31.4–39.2)
	Very satisfied	659/1650	17556/44281	39.6 (35.6–43.7)
	Not well satisfied^†^	398/1650	11102/44281	25.1 (21.5–28.6)
	Somewhat satisfied	261/1650	7084/44281	16.0 (13.1–18.9)
	Mixed or unsure	68/1650	2370/44281	5.4 (3.4–7.3)
	Somewhat dissatisfied	31/1650	767/44281	1.7 (0.7–2.8)
	Very dissatisfied	25/1650	656/44281	1.5 (0.6–2.3)
	Extremely dissatisfied	13/1650	225/44281	0.5 (0.1–1.1)

*Counts and percentages reflect values prior to imputation.

†‘Well Satisfied’ and ‘Not Well Satisfied’ is a dichotomous variable where ‘Well Satisfied’ includes both ‘Extremely’ and ‘Very Satisfied’ and ‘Not Well Satisfied’ includes the response categories ‘Somewhat Satisfied’ through ‘Extremely Dissatisfied’.

### Self-reported IPV

Table 3 shows the self-reported prevalence of a broad range of forms of IPV over the course of the current relationship. Perpetration of any form of physical and/or sexual abuse was seen in 9.4% (CI = 7.0 – 11.9%) of the population; victimization of any physical and/or sexual abuse was seen in larger fraction (15.4%, CI = 12.3 – 18.5%). With respect to specific acts, less physically injurious forms predominated (e.g., slapped) over more severe forms (kicked, bitten, or hit with fist). The most physically injurious forms (hit with something, beaten, choked, threatened with a gun or knife, forced sexual activity) occurred so infrequently (less than 20 observations) that reliable prevalence estimates are not possible. For some acts (e.g., pushed, grabbed, or shoved), rates of victimization and perpetration were similar; for others (e.g., slapped), victimization was much more prevalent than perpetration. For no act was perpetration more prevalent than victimization. No meaningful gender-specific differences in prevalence rates were seen for individual acts of physical or sexual IPV.

**Table 3:** Self-reported intimate partner violence by sex

		**Perpetration**		**Victimization**	
**Type**	**Sex**	**N (unweighted / weighted)**	**% (95% CI) weighted**	**N (unweighted / weighted)**	**% (95% CI) (weighted)**
Physical and sexual IPV					
Threatened to hit with fist or anything else which could hurt	Male	37/1871	4.5 (2.7–6.4)	40/2377	5.8 (3.6–8.0)
	Female	25/230	4.0 (2.2–5.8)	---	---
Thrown anything that could hurt	Male	---	---	54/3115	7.5 (5.1–10.0)
	Female	22/242	4.2 (2.2–6.2)	---	---
Pushed, grabbed, or shoved	Male	44/2655	6.5 (4.1–8.8)	41/2584	6.3 (3.9–8.7)
	Female	---	---	29/297	5.2(3.0–7.3)
Slapped	Male	---	---	70/3847	9.3 (6.5–12.1)
	Female	30/3000	5.2 (3.1–7.3)	---	---
Kicked, bit or hit with fist	Male	---	---	40/2130	5.2 (3.1–7.3)
	Female	---	---	---	---
Any physical and/or sexual IPV*	Male	72/3990	9.5 (6.7–12.2)	123/6910	16.4 (12.9–20.0)
	Female	57/525	9.0 (6.4–11.7)	47/437	7.5 (5.0–10.0)
	Overall	129/4515	9.4 (7.0–11.9)	170/7347	15.4 (12.3–18.5)
Emotional and financial IPV					
Limit contact with friends and family	Male	---	---	57/2471	6.0 (3.9–8.2)
	Female	---	---	---	---
Put downs and name calling	Male	103/4575	11.2 (8.4–14.0)	114/4950	12.1 (12.2–15.0)
	Female	78/685	12.0 (9.0–14.9)	81/646	11.3 (8.5–14.0)
Jealousy about communication with other sex	Male	51/2895	7.0 (4.5–9.6)	136/6385	15.6 (12.2–18.9)
	Female	55/572	9.9 (7.1–12.8)	90/831	14.4 (11.2–17.6)
Demanded to know about ‘who and where’ at all times	Male	22/1581	3.8 (1.8–5.9)	66/3350	8.2 (5.5–10.9)
	Female	---	---	32/329	5.7 (3.5–7.9)
Damaged or destroyed possessions or property	Male	---	---	34/1866	4.6 (2.5–6.6)
	Female	---	---	21/234	4.1 (2.2–6.0)
Any emotional and/or financial IPV*	Male	167/8161	19.4 (15.7–23.1)	237/10753	25.6 (21.6–29.6)
	Female	121/1092	18.8 (15.2–22.3)	150/1279	22.0 (18.3–25.7)
	Overall	288/9253	19.3 (16.1–22.6)	387/12032	25.1 (21.6–28.7)

---indicates suppressed result due to cells with fewer than 20 unweighted observations. Physical and sexual IPV response categories not included in the table because there were fewer than 20 unweighted observations for both males and females, for both perpetration and victimization, include being: hit with something, beaten, choked, threatened with/to use a gun/knife, and forced into unwanted sexual activity; emotional and financial IPV response categories not included in the table include being: harmed, or threatened to harm someone close, prevented knowledge or access to family income. However, positive responses to these low-prevalence items were included in the relevant aggregate outcomes (e.g., “any physical and/or sexual IPV perpetration” included those who had perpetrated unwanted sexual activity).

*Summary estimate presented using imputed data.

Table 3 also shows that any emotional and/or financial abuse was more prevalent than any physical and/or sexual abuse (perpetration of emotional and/or financial abuse in 18.8% (CI = 15.2 – 22.3%) and victimization in 22.0% (CI = 18.3 – 25.7%). The most prevalent form of abuse in victims was showing jealousy about talking to other men or women; put downs/name calling was the most prevalent form of abuse perpetrated. As with physical and/or sexual abuse, some acts occurred so infrequently that stable prevalence rates cannot be reported. No meaningful gender-specific differences in prevalence rates were seen for individual acts of emotional or financial IPV.

### Coexistence of physical and/or sexual and emotional and/or financial abuse

**Figure 1:**
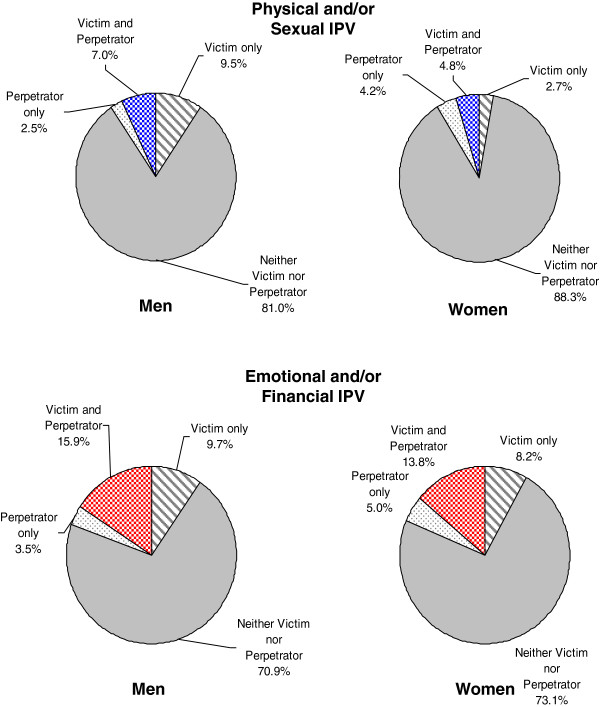
Mutuality of IPV in men and women.

**Figure 2:**
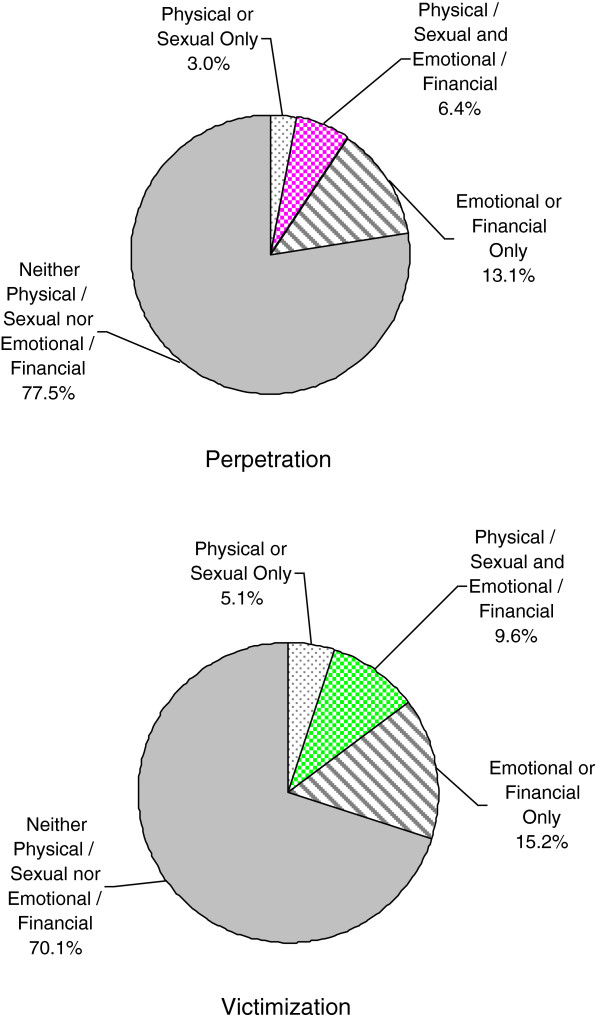
Co-existence of IPV types in perpetration and victimization.

Both physical and/or sexual abuse and emotional and/or financial abuse were commonly reciprocal, with 6.7% (CI = 4.6 – 8.8%) of individuals experiencing reciprocal physical and/or sexual abuse and a larger fraction of couples (15.7%, CI = 12.6 - 18.7%) experiencing reciprocal emotional and/or financial abuse. For emotional and/or financial abuse, the pattern of mutuality of abuse is similar in men and in women (Figure 1). However, for physical and/or sexual abuse, men were *more* likely to report being victims only (9.5%, CI = 6.6 - 12.3% vs. 2.7%, CI = 1.3 – 4.0). Physical and/or sexual and emotional and/or financial abuse commonly co-existed (6.4%, CI = 4.4 – 8.5%) for perpetration and 9.6% (CI = 7.0 – 12.1%) for victimization) (Figure 2).

### Other correlates of IPV perpetration and victimization

Tables 4, 5, 6, and 7 show the unadjusted associations between each of the IPV outcomes and socio-demographic variables, military characteristics, and psychosocial factors (e.g., probable depression). Among socio-demographic and military characteristics, few significant associations were found (men and married respondents both had a higher unadjusted risk of being physical and/or sexual IPV *victimization*). Remote deployment (more than two years previously vs. recent deployment) had an unadjusted association with physical and/or sexual perpetration IPV, while both remote deployment and never having been deployed (vs. recent deployment) was associated with physical and/or sexual IPV victimization. Remote deployment (vs. recent deployment) were also associated with both emotional and/or financial IPV perpetration and victimization.

**Table 4:** Risk factors for any physical and/or sexual IPV perpetration (N = 1745)*

** Characteristic**		**n/N (unweighted)**	**Weighted % (95% CI)**	**Unadjusted OR (95% CI)**	**p value**
Sex	Male	70/984	9.5 (6.7–12.3)	Ref	
	Female	55/709	9.0 (6.4–11.7)	0.94 (0.60–1.49)	0.81
Age	18–29	22/305	6.2 (1.5–11.0)	Ref	
	30–39	41/568	10.6 (6.0–15.2)	1.79 (0.69–4.59)	0.23
	40–60	62/820	10.3 (6.7–13.9)	1.74 (0.71–4.27)	0.23
Marital status	Married	95/1094	10.4 (7.3–13.5)	Ref	
	Not married	30/593	7.7 (3.6–11.7)	0.71 (0.37–1.38)	0.32
Relationship satisfaction	Well satisfied	65/1239	6.9 (4.3–9.6)	Ref	
	Not well satisfied	57/395	17.8 (11.7–24.0)	2.91 (1.62–5.24)	0.000
Element	Land	65/802	10.7 (6.8–14.6)	Ref	
	Sea	22/264	8.8 (3.8–13.8)	0.8 (0.38–1.70)	0.57
	Air	38/624	8.3 (4.4–12.2)	0.8 (0.39–1.50)	0.41
Rank	Junior NCM^†^	41/474	10.1 (6.0–14.1)	Ref	
	Senior NCM^†^	23/293	9.7 (5.0–14.4)	0.96 (0.48–1.93)	0.91
	Officer	61/926	7.8 (5.1–10.6)	0.76 (0.42–1.36)	0.35
Deployment	Recent	39/793	4.8 (2.6–7.1)	Ref	
History	Remote	46/457	14.5 (9.6–19.4)	3.34 (1.78–6.28)	0.00
	Never	40/442	6.3 (3.2 –9.4)	1.33 (0.65–2.72)	0.44
PTSD symptoms	No	104/1582	7.9 (5.7–10.2)	Ref	
	Yes	21/108	29.1 (13.3–45.0)	4.78 (2.09–10.93)	0.00
Probable depression	No	95/1522	8.1 (5.8–10.5)	Ref	
	Yes	23/120	28.2 (12.6–43.8)	4.41 (1.92–10.13)	0.00
High–risk drinking	No	85/1274	9.0 (6.2–11.8)	Ref	
	Yes	29/240	13.3 (6.4–20.3)	1.56 (0.78–3.12)	0.21

*All data reflect values prior to imputation.

†Junior NCM = non-commissioned member at rank below Sergeant or the equivalent; Senior NCM = non-commissioned member at rank of Sergeant (or the equivalent) or above.

**Table 5:** Risk factors for any physical and/or sexual IPV victimization (N = 1745)*

** Characteristic**		**n/N (unweighted)**	**Weighted % (95% CI)**	**Unadjusted OR (95% CI)**	**p value**
Sex	Male	119/988	16.2 (12.7–19.7)	Ref	
	Female	45/709	7.3 (4.9–9.7)	0.41 (0.26–0.63)	0.00
Age	18–29	29/306	13.0 (6.0–19.9)	Ref	
	30–39	56/570	17.4 (11.6–23.2)	1.42 (0.68–2.97)	0.35
	40–60	79/821	14.8 (10.6–19.0)	1.17 (0.58–2.36)	0.67
Marital status	Married	128/1098	17.8 (13.9–21.8)	Ref	
	Not married	36/593	10.0 (5.2–14.8)	0.51 (0.28–0.94)	0.03
Relationship satisfaction	Well satisfied	84/1242	11.8 (8.4–15.3)	Ref	
	Not well satisfied	75/395	24.3 (17.2–31.3)	2.39 (1.44–3.97)	0.001
Element	Land	87/805	14.8 (10.4–19.2)	Ref	
	Sea	28/262	17.0 (8.9–25.1)	1.18 (0.60–2.31)	0.63
	Air	49/627	14.7 (9.6–20.0)	1.00 (0.58–1.71)	0.99
Rank	Junior NCM^†^	54/473	15.8 (10.8–20.9)	Ref	
	Senior NCM^†^	28/295	16.0 (10.0–22.0)	1.01 (0.56–1.83)	0.97
	Officer	82/929	12.6 (9.0–16.3)	0.77 (0.46–1.27)	0.31
Deployment history	Recent	59/795	7.7 (4.8–10.6)	Ref	
	Remote	58/455	19.5 (14.0–25.0)	2.92 (1.70–5.00)	0.00
	Never	47/446	14.9 (9.3–20.5)	2.10 (1.15–3.85)	0.02
PTSD symptoms	No	143/1585	14.2 (11.1–17.3)	Ref	
	Yes	21/109	28.0 (12.5–43.5)	2.4 (1.05–5.31)	0.04
Probable depression	No	138/1524	14.4 (11.2–17.6)	Ref	
	Yes	20/121	26.0 (10.6–41.4)	2.09 (0.90–4.86)	0.09
High-risk drinking	No	111/1275	14.9 (11.3–18.5)	Ref	
	Yes	39/241	22.3 (13.4–31.3)	1.64 (0.91–2.96)	0.10

*All data reflect values prior to imputation.

†Junior NCM = non–commissioned member at rank below Sergeant or the equivalent; Senior NCM = non-commissioned member at rank of Sergeant (or the equivalent) or above.

**Table 6:** Risk factors for any emotional or financial IPV perpetration (N = 1745)*

** Characteristic**		**n/N (unweighted)**	**Weighted % (95% CI)**	**Unadjusted OR (95% CI)**	**p value**
Sex	Male	161/985	19.4 (15.7–23.1)	Ref	
	Female	118/709	18.7 (15.1–22.2)	0.95 (0.68–1.33)	0.78
Age	18–29	48/306	16.5 (9.0–24.1)	Ref	
	30–39	102/567	21.6 (15.7–27.5)	1.39 (0.72–2.67)	0.32
	40–60	129/821	19.2 (14.7–23.7)	1.20 (0.64–2.23)	0.57
Marital status	Married	201/1096	20.3 (16.4–24.3)	Ref	
	Not married	77/591	17.3 (11.5–23.1)	0.81 (0.51–1.31)	0.41
Relationship satisfaction	Well satisfied	149/1240	14.3 (10.8–17.9)	Ref	
	Not well satisfied	122/394	34.9 (27.1–42.7)	3.21 (2.05–5.04)	0.000
Element	Land	140/801	20.1 (15.2–24.9)	Ref	
	Sea	47/264	20.2 (12.1–28.4)	1.01 (0.56–1.82)	0.97
	Air	92/625	18.0 (12.6–23.3)	0.87 (0.54–1.40)	0.57
Rank	Junior NCM^†^	82/471	18.3 (13.1–23.6)	Ref	
	Senior NCM^†^	58/295	22.6 (16.1–29.2)	1.31 (0.78–2.18)	0.31
	Officer	139/928	17.0 (13.3–20.8)	0.92 (0.59–1.42)	0.70
Deployment history	Recent	110/797	13.7 (10.0–17.3)	Ref	
	Remote	98/451	24.0 (18.3–29.6)	1.99 (1.28–3.09)	0.00
	Never	71/445	17.4 (11.4–23.5)	1.33 (0.79–2.24)	0.28
PTSD symptoms	No	242/1582	17.8 (14.5–21.0)	Ref	
	Yes	37/109	38.8 (22.5–55.1)	2.93 (1.43–6.02)	0.00
Probable depression	No	226/1525	17.1 (13.8–20.4)	Ref	
	Yes	39/120	38.1 (22.8–53.5)	2.99 (1.50–5.96)	0.00
High–risk drinking	No	198/1275	18.1 (14.4–21.8)	Ref	
	Yes	59/239	32.7 (22.9–42.5)	2.20 (1.32–3.67)	0.00

*All data reflect values prior to imputation.

†Junior NCM = non-commissioned member at rank below Sergeant or the equivalent; Senior NCM = non-commissioned member at rank of Sergeant (or the equivalent) or above.

**Table 7:** Risk factors for any emotional or financial IPV victimization (N = 1745)*

** Characteristic**		**n/N (unweighted)**	**Weighted % (95% CI)**	**Unadjusted OR (95% CI)**	**p value**
Sex	Male	228/985	25.3 (21.3–29.3)	Ref	
	Female	146/710	21.8 (18.2–25.5)	0.82 (0.61–1.12)	0.21
Age	18–29	65/305	21.9 (13.5–30.2)	Ref	
	30–39	130/569	29.0 (22.4–35.6)	1.46 (0.82–2.62)	0.20
	40–60	179/821	23.7 (19.0–28.4)	1.11 (0.64–1.93)	0.71
Marital status	Married	257/1096	23.8 (19.7–27.9)	Ref	
	Not married	115/592	27.0 (20.2–33.7)	1.18 (0.78–1.78)	0.42
Relationship satisfaction	Well satisfied	183/1239	17.5 (13.8–21.3)	Ref	
	Not well satisfied	173/396	46.2 (38.1–54.3)	4.04 (2.66–6.13)	0.000
Element	Land	175/800	23.4 (18.3–28.4)	Ref	
	Sea	73/265	30.2 (21.1–39.3)	1.42 (0.85–2.38)	0.18
	Air	124/626	24.2 (18.3–30.0)	1.04 (0.68–1.60)	0.84
Rank	Junior NCM^†^	102/472	24.8 (18.9–30.7)	Ref	
	Senior NCM^†^	69/295	25.0 (18.4–31.5)	1.01 (0.63–1.62)	0.97
	Officer	203/928	25.0 (20.6–29.4)	1.01 (0.68–1.50)	0.96
Deployment history	Recent	156/797	19.6 (15.4–23.9)	Ref	
	Remote	121/454	29.4 (23.3–35.5)	1.70 (1.14–2.54)	0.01
	Never	97/443	22.9 (16.4–29.4)	1.21 (0.77–1.91)	0.41
PTSD symptoms	No	332/1583	23.2 (19.6–26.7)	Ref	
	Yes	42/109	47.2 (30.6–63.7)	2.96 (1.48–5.91)	0.00
Probable depression	No	307/1524	22.5 (18.9–26.0)	Ref	
	Yes	50/121	48.8 (33.4–64.3)	3.30 (1.72–6.34)	0.00
High-risk drinking	No	258/1273	22.7 (18.7–26.7)	Ref	
	Yes	85/240	41.0 (31.0–51.1)	2.37 (1.47–3.81)	0.00

*All data reflect values prior to imputation.

†Junior NCM = non-commissioned member at rank below Sergeant or the equivalent; Senior NCM = non-commissioned member at rank of Sergeant (or the equivalent) or above.

While relationship dissatisfaction had an unadjusted relationship with all four outcomes, most individuals in relationships in which IPV had occurred were nevertheless satisfied with their relationship. For example, 59.1% (CI = 48.0 – 70.3%) of victims of any physical and/or sexual abuse were well-satisfied with their relationship (versus 77.6%, CI = 73.9 – 81.2%, for non-victims). PTSD symptoms were associated with all outcomes (unadjusted OR ranging from 2.4 to 4.8); probable depression was associated with all but one (OR range 2.1 – 4.4); high risk drinking was associated with both forms of emotional and/or financial IPV (OR = 2.2 for perpetration and 2.4 for victimization).

The four separate logistic regression models are shown in Table 8. Two of the initial models showed poor model fit, but subsequent exclusion of age in one model and rank in another yielded adequate fit; neither of these variables had an unadjusted association with the dependent variable in the model. Sex was independently associated only with physical and/or sexual abuse victimization, with females having a *lower* risk than males (adjusted OR = 0.39, CI = 0.24 – 0.65). Those who were not married had a lower risk of physical and/or sexual victimization compared to those in a marital intimate relationship (OR = 0.45, CI = 0.23 – 0.89). Relationship dissatisfaction was independently associated with all four outcomes (OR range from 2.3 to 3.7). PTSD symptoms were independently associated only with physical and/or sexual abuse perpetration (OR = 3.2, CI = 1.4 – 7.9); probable depression retained its unadjusted relationship with physical and/or sexual IPV perpetration, emotional and/or financial IPV perpetration, and emotional and/or financial IPV victimization (OR range from 2.5 to 2.7). High risk drinking was independently associated with emotional and/or financial abuse victimization (OR = 2.0, CI = 1.2 – 3.4) and with perpetration as well (OR = 2.0, CI = 1.1 – 3.6). Tests for multicollinearity and model fit were reassuring for all final models.

**Table 8:** Logistic regression models by abuse type

		**Physical/sexual**		**Emotional/financial**	
**Variable**		**Perpetration N = 1729**	**Victimization N = 1729**	**Perpetration N = 1729**	**Victimization N = 1729**
		**Adjusted OR (95% CI)**	**Adjusted OR (95% CI)**	**Adjusted OR (95% CI)**	**Adjusted OR (95% CI)**
Age					
	18–29	Ref	---	Ref	Ref
	30–39	1.61(0.62–4.18)	---	1.19 (0.59–2.37)	1.43 (0.76–2.69)
	40–60	1.35 (0.56–3.22)	---	0.87 (0.43–1.75)	0.99 (0.53–1.86)
Sex					
	Male	Ref	Ref	Ref	Ref
	Female	0.91 (0.53–1.57)	**0.39 (0.24–0.65)**^†^	0.99 (0.67–1.47)	0.78 (0.54–1.11)
Marital status					
	Married	Ref	Ref	Ref	Ref
	Not married	0.70 (0.36–1.39)	0.45 (0.23–0.89)*	0.73 (0.43–1.22)	1.12 (0.71–1.76)
Relationship satisfaction					
	Well satisfied	Ref	Ref	Ref	Ref
	Not well satisfied	**2.81 (1.55–5.10)**^†^	2.32 (1.35–3.97)*	**3.10 (1.94–4.94)**^†^	**3.72 (2.42–5.72)**^†^
Element					
	Land	Ref	Ref	Ref	Ref
	Sea	0.61 (0.27–1.36)	0.86 (0.44–1.67)	0.75 (0.39–1.42)	1.19 (0.68–2.08)
	Air	0.83 (0.42–1.62)	0.89 (0.48–1.66)	0.90 (0.55–1.49)	1.16 (0.73–1.85)
Rank					
	Junior NCM^†^_†_	---	Ref	Ref	Ref
	Senior NCM^†^_†_	---	0.83 (0.44–1.57)	1.49 (0.82–2.72)	1.03 (0.59–1.80)
	Officer	---	0.64 (0.38–1.08)	0.94 (0.58–1.53)	1.11 (0.71–1.75)
Deployment history					
	Recent	Ref	Ref	Ref	Ref
	Remote	**3.39 (1.67–6.89)**^†^	**2.86 (1.62–5.07)**^†^	2.01 (1.23–3.26)*	1.78 (1.13–2.80)*
	Never	1.96 (0.85–4.49)	2.63 (1.30–5.30)*	1.77 (0.97–3.21)	1.41 (0.83–2.42)
PTSD symptoms					
	No	Ref	Ref	Ref	Ref
	Yes	3.28 (1.36–7.92)*	1.89 (0.77–4.65)	1.81 (0.73–4.50)	1.93 (0.89–4.17)
Probable depression					
	No	Ref	Ref	Ref	Ref
	Yes	2.73 (1.19–6.28)*	1.54 (0.55–4.33)	2.52 (1.05–6.05)*	2.47 (1.18–5.18)*
High-risk drinking					
	No	Ref	Ref	Ref	Ref
	Yes	1.32 (0.60–2.91)	1.50 (0.77–2.92)	2.00 (1.12–3.56)*	2.01 (1.17–3.43)*
		F(12, 1713) = 3.72, p < 0.0000	F(12, 1715) = 3.72, p < 0.0000	F(14, 1715) = 3.82, p < 0.0000	F(14,1714) = 5.24, p < 0.0000

**Bold** indicates statistically significant findings (p < 0.05).

*p < 0.05.

†p ≤ 0.001.

‡Junior NCM = non-commissioned member at rank below Sergeant or the equivalent; Senior NCM = non-commissioned member at rank of Sergeant (or the equivalent) or above.

---indicates that variable excluded because inclusion resulted in poor model fit indices.

The unadjusted association seen between remote deployment (vs. recent deployment) and all four IPV outcomes remained significant after adjusting for potential confounders (OR range from 1.8 to 3.4). The association between physical and/or sexual IPV victimization and never having deployed (vs. recent deployment) also remained after adjustment (OR = 2.6, CI = 1.3 – 5.3).

## Discusssion

### Summary of key findings

Self-reported physical and/or sexual IPV perpetration and victimization were reported in an important minority of Canadian Armed Forces personnel over the course of their current relationship: 9% reported having perpetrated physical and/or sexual IPV and 15% reported having been a victim of it. Less physically injurious forms of abuse predominated, and the frequencies of more physically injurious forms were low enough that their prevalence could not be estimated with confidence. Emotional/financial abuse was more prevalent, with 19% reporting perpetration and 22% reporting victimization. Physical/sexual abuse often co-existed with emotional and/or financial abuse, and perpetration and victimization were often mutual.

With respect to independent risk factors, men and married respondents had a higher risk of physical and/or sexual *victimization*. Very few other gender-related differences were seen, although power to detect such differences was low. Relationship dissatisfaction was associated with all abuse outcomes, but most of those in relationships where abuse had occurred were still satisfied with their relationship. Probable depression had a strong, independent relationship with every abuse outcome except for physical and/or sexual victimization. While PTSD symptoms had a unadjusted relationship with all four outcomes, modeling only confirmed one significant multivariate relationship (with physical and/or sexual IPV perpetration). High-risk drinking was associated with emotional and/or financial abuse perpetration and victimization. Deployment was associated with all four IPV outcomes, and the timing of deployment appeared to be a relevant factor: Remote deployment was an independent risk factor for all four IPV outcomes. Never having deployed was an independent risk factor for physical and/or sexual IPV victimization.

### Comparison with other relevant literature

#### Prevalence rates

Comparing IPV prevalence rates across studies is problematic: Different surveys use different IPV assessment items, different survey modes (e.g., telephone vs. paper), and different recall periods. All of these can have an effect on prevalence rates. Some surveys ask about IPV in the context of the current relationship, while others ask about IPV in *any* intimate relationship. Socio-demographic differences between populations are often present, but stratifications that would permit careful adjustment are usually not reported.

The most recent Canadian general population data on self-reported IPV victimization come from Statistics Canada’s 2009 General Social Survey (GSS) on victimization [2]. This telephone survey used the same instrument that we used for assessing IPV victimization, but the primary recall period was five years in the context of both present and past intimate relationships. The inclusion of ex-intimate partners in the GSS but not our survey is important because they account for a disproportionate fraction of IPV [2]. There are also important differences between the military population (largely younger males, a group known to be at increased risk for IPV perpetration [1,5]) and the GSS frame (the broader Canadian general population).

Keeping in mind these difficulties, the 2009 GSS identified self-reported physical and/or sexual IPV victimization in 6% over the previous five years compared to 15% over the lifetime of the current relationship used in our study. Self-reported emotional and/or financial IPV victimization by the current or ex-intimate partner over the previous five years was seen in 17% of the GSS population, compared to 25% of the CAF population by the current spouse over the lifetime of their relationship. The most that can be taken away from this comparison, given the important differences between the studies and their populations, is that their rates of IPV victimization are unlikely to be dramatically higher or dramatically lower than that of the comparable Canadian general population. The 2009 GSS did not assess IPV perpetration.

The most recent (2010) large survey of IPV in the US was the National Intimate Partner and Sexual Violence Survey (NISVS) [36]. Using a similar inventory of physical IPV victimization items, 5% of men and 4% of women reported physical IPV victimization over the previous 12 months with any intimate partner; lifetime prevalence rates were 33% and 28% for men and women, respectively [36]. Using a much broader range of emotional and/or financial IPV victimization items, 12-month and lifetime prevalence rates for any abuse were 18% and 49% for men, respectively and rates for women were 14% and 48%, respectively [36]. As with the 2009 GSS data, the most that can be drawn from this comparison to our findings is that they are not dramatically different when important differences in the studies are considered. The NISVS did not assess IPV perpetration.

Similar issues arise in comparing CAF prevalence rates with rates in the US military. The most comparable data comes from a 2006 survey of US Air Force personnel [37]. This survey used analogous data from military spouses to adjust for the significant underreporting of both victimization and perpetration. Unadjusted (hence more comparable) 12-month rates of IPV perpetration and victimization in male service members were 5.5% and 14.5%; corresponding rates in female service members were 9.1% and 11.3%. Unfortunately, this study used a much more detailed inventory of abusive acts, which presumably would lead to higher prevalence rates for aggregate variables for “any IPV.” Again, the most that can be said is that CAF rates are not dramatically different from these, given the important differences between the studies.

#### Risk factors

A broad range of risk factors have been reported for physical and/or sexual IPV perpetration in other studies [1,5]. While physical and/or sexual IPV victimization is overall similar in men and women or (counter-intuitively) more prevalent in *men*[36] (as we also found), men are much more likely to inflict sexual violence and more physically injurious forms of physical IPV and less likely to inflict less physically injurious forms [1,5]. We could not assess gender differences in more severe forms of IPV because of their low prevalence and our modest sample size. Others have typically found a greater risk of IPV in non-marital intimate relationships [2]; in contrast, we found largely no relationship except for physical and/or sexual IPV victimization in which we found a *greater* risk in marital relationships. A possible explanation for this discrepancy could be a confounding effect of the variable recall period for IPV. That is, married individuals may have had longer relationships hence a longer effective recall period for IPV. Youth is also a well-recognized risk factor for IPV perpetration and victimization [1,5], but we did not find such an association. In military populations, lower rank has been shown to be a risk factor for all forms of IPV [37,38], at least for recent abuse. We saw no such association with rank; our use of a presumably longer recall period may account for this.

The association of probable depression with IPV *victimization* we found is both sensible and well-documented in the literature [39,40]; we did not, however, find the expected association between PTSD symptoms and IPV victimization. The predominance of males in our sample and of females in other work on PTSD and victimization may account for this difference. Data on the association of PTSD and depression with perpetration are much sparser, and the limited population-based data focuses on male perpetrators. Both conditions can be associated with anger, irritability, emotional dysregulation, and difficulties in interpersonal relationships, all of which are plausible contributors to IPV perpetration [6,41-46]. Consistent with this, clinical samples of veterans seeking care for PTSD reveal high rates of IPV perpetration [47]. Depression has also been shown to be a risk factor for IPV perpetration in military personnel [42,48,49]. Our findings on the association of high risk drinking and emotional and/or financial IPV are consistent with other literature [50]; our failure to confirm the known association of high-risk drinking and physical and/or sexual IPV perpetration in military populations [51] may again relate to the presumably longer recall period we used for IPV and the preponderance of high risk drinking among younger personnel [10]. That is, alcohol use may have contributed to IPV when it occurred, which might have been in the distant past. Many studies of military personnel and others have confirmed the self-evident relationship between relationship dissatisfaction and IPV [7].

The association of deployment with IPV perpetration in military personnel and veterans has been inconsistent, with some studies showing no apparent association and others showing a small association. Combat exposure in particular has been shown to be associated with perpetration of IPV, and this appears to be largely mediated by PTSD symptoms [7]. Our findings show a strong and consistent independent association of remote deployment (vs. recent deployment) with all four IPV outcomes, even after adjustment for mental health problems and high-risk drinking. To the best of our knowledge, no other study has shown an increased risk of emotional and/or financial IPV victimization (and not other forms of IPV) in those who never deployed (vs. recent deployers). We are not aware of any other work on the association of deployment with IPV *victimization.*

In summary, the risk factors we identified are plausible and largely consistent with other military and civilian studies or explainable by differences in methods or study populations. While the observed association of IPV with remote deployments is consistent with some other studies, it should still be interpreted with caution in light of the limitations laid out below.

### Limitations

The most important limitation of this study is that it relies on self-report of both victimization and perpetration. Both are subject to substantial under-reporting [37]. However, this limitation applies to nearly all prevalence research in the field. The response rate for this survey (53%) is low enough that response bias is possible, and this could have an important impact on the estimated prevalence rates and the associations we found, and the direction and magnitude of possible bias are unknown. However, our response rate was similar to those of other large, military IPV surveys such as Foran et al.’s USAF survey (45% [37]). Even national civilian surveys on IPV see low response rates (e.g., 62% for the Canadian 2009 GSS [2]) and low contact rates (approximately 30% for the 2012 US NISVS [36]). In addition, victims of IPV characterized by power and control motives (so-called “intimate terrorism”) may be underrepresented among respondents who fear reprisal [52].

The second most important limitation of the study relates to the recall period used for IPV (over the life of the current relationship): This is problematic for several reasons: First, we did not assess the duration of the current relationship, so comparison with studies with other recall periods is impossible. However, the age of the study population and collateral information on relationship stability in the CAF lead us to believe that the average duration is well above the five years used in the 2009 GSS [2]. Prevalence rates are sensitive to the recall period: For example, in the 2009 GSS, the five year prevalence rate for any physical and/or sexual abuse was 6%, but the one year prevalence was only 2%. Prolonged recall periods are likely to be prone to recall bias for events that may have been in the distant past. Notwithstanding our likely prolonged recall period, the events of greatest interest (the most physically injurious forms of physical and sexual IPV) occurred infrequently enough that we could not estimate their prevalence reliably. The recall period also leads to uncertainty about the temporality of IPV relative to deployment and hence complicates the interpretation of the observed association with deployment.

We did not assess the frequency or perceived impact of acts of abuse, so it is possible that the events documented occurred only once (and potentially many years previously). The recall period and failure to assess IPV frequency or impact may account for the substantial relationship satisfaction in most couples in which IPV had occurred. Limiting the reporting of IPV to only the current relationship may have introduced bias, while at the same time eroding comparability with other surveys [2]. While IPV items we used were taken from a series of large, national Canadian surveys [2], the items were selected and adapted from a longer instrument [27], meaning that bias could have been introduced.

Our analysis draws on cross-sectional data, so the causal nature of the association between abuse and the co-variates we measured is uncertain. This is particularly problematic for mental disorders and relationship dissatisfaction, for which bi-directional causation is highly plausible. Bi-directional causation is also probable for mental disorders and IPV itself, and the frequently reciprocal nature of IPV adds another layer of complexity to teasing apart what is a cause and what is an effect. Nevertheless, while the etiological role of mental disorders, high-risk drinking, and relationship satisfaction in active IPV is unclear due to the cross-sectional nature of the study and mismatch between the relevant recall periods, it is clear that current or recent mental health problems, high-risk drinking, and relationship dissatisfaction do help identify families with an elevated risk for past or present IPV.

Our modest sample size may have limited our ability to detect some small but important differences. In particular, the smaller numbers of women in the sample precluded in-depth exploration of gender differences; this was not, however, a primary goal of this paper. The preponderance of less severe forms of IPV in our sample and the use of aggregate outcomes mean that our findings are driven by the more common, less injurious forms of IPV rather than those of greatest clinical significance. This limitation is inherent in all but the largest IPV surveys: The IPV captured (both perpetretaion and victimization) largely reflects “common” or “situational” IPV in which patriarchal power and control motives are not prominent [53]. It is conceivable that the covariates of interest have a different relationship with these importantly different forms of IPV. Our use of a likely prolonged recall period likely magnified this focus in that we presumably captured many cases of isolated instances in the distant past.

The modest sample size, low prevalence of more severe forms of IPV, and use of aggregate outcomes lumping more and less severe forms of IPV (in which gender differences are prominent [54]) also limited our ability to explore important gender differences in IPV. Similarly, we were unable to explore the consequential motivations underlying bi-directional (mutual) IPV (e.g., whether it represents a case of mutual aggression or of unilateral aggression and subsequent self-defense [54]).

The recall period for mental disorders and for alcohol abuse was short relative to that for IPV (one to 12 months vs. the life of the current relationship). In addition, the brief survey instruments we used for assessment of mental disorders certainly resulted in some misclassification. We were able to exclude the possibility that the association of remote deployment with IPV was fully mediated by *current* or *recent* symptoms of PTSD, depression, or alcohol misuse. However, the lack of detail on the temporality of deployment and IPV events and the lack of information on *past* mental disorders (including alcohol abuse) means that we cannot exclude the possibility of mediation of past IPV by more remote deployment-related mental disorders.

The association between remote deployment and IPV may be due to residual confounding: Deployment is not a random event in military organizations: Some military occupations are more likely to deploy (and to do so repetitively) than others. Military occupations are highly diverse, encompassing physicians, administrators, and cooks in addition to infanteers, boatswains, and fighter pilots; each likely has its own profile of personality characteristics, socioeconomic backgrounds, and other individual differences of potential importance to the genesis of IPV. Personnel are selected for deployment based on work performance, medical fitness, and family readiness, all of which could have plausible associations with factors that drive IPV.

Our measurement of deployment status was crude: If perpetration of post-deployment IPV is mediated by traumatic deployment experiences and post-deployment mental disorders, our deployed group was likely heterogeneous enough that we could have missed an important subgroup (e.g., recent Afghanistan returnees with heavy combat exposure) at increased risk for IPV. The first two years after return from deployment is a time of significant change for military personnel and their families, as service members adapt to the home environment, re-establish relationships, recover (if needed) from deployment related psychological disorders, and prepare for the next deployment [55]. As such, our definition of “recent deployment” as being within the previous two years may have obscured important temporal effects of deployment on IPV. The limitations in using such measures for deployment history may account for the inconsistent relationship seen between it and IPV in other studies [7].

### Implications

Public concern about IPV in military families has focused on male military personnel inflicting severe forms of PTSD-fueled physical violence on their female civilian intimate partners soon after return from difficult combat deployments. However, our data paint a far more intricate picture of IPV in CAF families: Male and female CAF personnel are both victims and perpetrators of the full spectrum of physical and psychological IPV, which often co-exist and occur reciprocally. While less physically injurious forms of IPV predominate, their substantial prevalence may drive a sizable public health impact.

These findings all argue for a broad focus on the full spectrum of IPV in our prevention efforts in CAF families. The gross similarity between IPV and its correlates in CAF personnel and those in the general population and in other military populations suggest that prevention efforts effective in those other contexts [5] are the most promising ones for the CAF. Unfortunately, the interventions having the strongest evidence of effectiveness (e.g., school-based programs for prevention of dating violence in adolescents) are not well-suited to the CAF’s context [9].

PTSD symptoms were indeed associated with perpetration of physical and/or sexual IPV, but it appears to be only one factor among many; probable depression had a broader association with different IPV outcomes. Depression and PTSD have different symptom profiles and hence different potential effects on behaviour. As well, each is expected to have different causal linkages with perpetration vs. victimization of IPV. As such, the differences in their associations with different forms of IPV are unsurprising.

The higher prevalence of depression vs. PTSD in the CAF [56], its plausible aetiological linkage with IPV, and its broader association with different forms of IPV argue for ongoing efforts in overcoming barriers to mental health care for depression and improving the quality of care delivered. Such efforts are essential in identified IPV cases as part of rehabilitation and prevention of recurrence. In addition, improvement of population mental health may have primary prevention effects for IPV. This strong and consistent interrelationship between IPV and mental disorders also argues for careful assessment of mental disorders in those touched by IPV and, conversely, the careful assessment of IPV in those with mental disorders. All of these same considerations apply to the relationship between high-risk drinking and IPV.

The association of IPV with remote as opposed to recent deployment is evidence against the hypothesis that failure to adapt combat behaviours is a common mechanism for post-deployment IPV perpetration. Hence, the benefits of efforts to help personnel adapt these behaviours as they transition home after deployment [22] will likely lie elsewhere. This also suggests that the immediate post-deployment period is not one of significantly increased risk for any form of IPV: Indeed, the prevalence of all forms of IPV was *lowest* in recent deployers. Analogously, while remote deployment was independently associated with all forms of IPV, the differences between the prevalence in remote vs. never deployers were largely small. This argues for targeting the broader military population for preventive interventions as opposed to focusing just on deploying personnel. That said, the deployment-related prevalence difference was greatest for physical and/or sexual IPV perpetration, which has been the type of IPV that has been of greatest public concern in military families. Hence, further research in this area is warranted.

We do not have a clear explanation for the strong and consistent independent association of remote deployment and all IPV outcomes except to say that in the context of the important limitations discussed above, it should *not* be interpreted as clear evidence that deployment is an important etiological factor in IPV in military populations. In other words, this association tells us far more about the limitations of our methods than it does about the contribution of remote deployments to IPV. A deeper understanding of this observed association will demand future studies that do a better job establishing the temporality of the most relevant events (deployment, mental disorder onset, and IPV); this will be difficult without longitudinal data collection. Distinguishing between “difficult” deployments characterized by a high risk of PTSD and depression (and hence potentially increased risk of IPV) and “less difficult” ones will be essential—ideally, individual assessment of the extent of exposure to combat and other forms of adversity will be included. Finally, better controlling for individual differences between those who happen to deploy and those who do not will minimize the risk of residual confounding.

While more research on the relationship between deployment and IPV is required, we believe that it is unlikely that deployment effects large enough to drive major differences in prevention or treatment programs will be found. It should be kept in mind that IPV is prevalent in non-military families as well; as such it cannot be largely a consequence of military life. While the impact of efforts to mitigate work-family conflict in military families on incidents of IPV is speculative, efforts in these areas have intrinsic value and should be pursued on that basis.

Research on victimization in military intimate partners is also essential, given the underreporting of perpetration. The perhaps surprisingly substantial prevalence of victimization among male military personnel means that understanding their perpetrating intimate partners will aid prevention and control efforts. More useful than precise prevalence estimates will be contextual information about IPV in military families, such as impact, use of services, and barriers to services use. In particular, the role that the involvement of military police and the chain of command plays as a potential barrier to reporting and care needs exploration [9]. Conversely, the role that such involvement can have in facilitating prevention merits attention. Finally, understanding the prevailing attitudes about IPV in military families would help in development of social marketing and educational programs. Until such time as this information is available, the best assumption about these attitudes is that they are similar to those in the Canadian general population [57].

## Conclusion

Intimate partner violence is a part of the fabric of intimate relationships for an important minority of CAF families, as it is in the Canadian general population. Assuming that data on the consequences of IPV in the general population also apply to CAF families, the full spectrum of IPV will be associated with impacts of interest to the CAF as an employer, as a provider of health services, as a public health system, and as a provider of services and benefits to family members. Additional research on military-specific aspects of IPV is needed, but meaningful prevention efforts (such as those recommended by the recent CAF Expert Panel on the Prevention of Family Violence, [9]) can take place as additional data accumulates.

Relative to other employers, military organizations are in an enviable position to engage in prevention of intimate partner violence. They typically deliver, manage, and pay for health services for their employees, and, depending on the nation, for their families as well. Military families have a strong attachment to the military employer, and many receive services and support (e.g, child care, counselling, educational programming) through military family services organizations. Military police activities can be readily coordinated with other prevention and intervention approaches. Optimal leadership and unit climate is another potential point of leverage [49], and the potential effect of IPV perpetration on continued military service may serve as a potent deterrent to recidivism [58]. Military organizations can and do require resilience training and training on intimate partner violence for their employees. They also have a robust public health capacity for surveillance. Moreover, military organizations have the ability to bring all of these prevention efforts to bear in a coordinated way. Finally, military organizations stand to reap the benefits of effective prevention of intimate partner violence.

We did not find evidence of an epidemic of perpetration of severe forms of physical abuse by recently deployed male personnel on their civilian intimate partners. Nor did we find evidence to suggest that the face of IPV is dramatically different in the CAF than in comparable civilian populations. However, these conclusions need to be considered in light of the important limitations of the study. Regardless, as additional research on IPV accumulates to address these limitations, it makes sense to focus not on special risks of IPV or special barriers to its prevention in the CAF, but instead on special *opportunities* for prevention offered by the military environment. Above all else, IPV in military organizations needs to be addressed as a *public* health problem, not a *deployment* health problem.

## Abbreviations

CAF: Canadian armed forces; CI: Confidence interval; CIDI-SF: Composite international diagnostic interview short-form; GSS: General social survey; HLIS: Health and lifestyle information survey; IPV: Intimate partner violence; NCM: Non-commissioned member; OR: Odds ratio; NISVS: National intimate partner and sexual violence survey; PTSD: Post-traumatic stress disorder; US: United States of America.

## Competing interests

The authors declare that they have no competing interests.

## Authors’ contributions

MZ contributed to the study design and the analytical approach, contributed to the interpretation, and wrote most of the Background, Discussion, and Conclusions sections. MW contributed to the study design and analytical approach, performed the analyses, contributed to the interpretation of the results, and wrote most of the Methods and Results sections. Both authors have read and approved the final manuscript.

## Pre-publication history

The pre-publication history for this paper can be accessed here:

http://www.biomedcentral.com/1471-2458/13/1019/prepub
